# Integrating omics reveals that miRNA-guided genetic regulation on plant hormone level and defense response pathways shape resistance to *Cladosporium fulvum* in the tomato *Cf-10-*gene-carrying line

**DOI:** 10.3389/fgene.2023.1158631

**Published:** 2023-05-25

**Authors:** Guan Liu, Fengjiao Liu, Dongye Zhang, Tingting Zhao, Huanhuan Yang, Jingbin Jiang, Jingfu Li, He Zhang, Xiangyang Xu

**Affiliations:** ^1^ College of Advanced Agriculture and Ecological Environment, Heilongjiang University, Harbin, China; ^2^State Key Laboratory of Tree Genetics and Breeding, College of Forestry, Northeast Forestry University, Harbin, China; ^3^ College of Horticulture and Landscape Architecture, Northeast Agricultural University, Harbin, China

**Keywords:** tomato, *Cladosporium fulvum*, *Cf-10* gene, miRNA, gene expression regulation, defense response

## Abstract

Invasion of *C. fulvum* causes the most serious diseases affecting the reproduction of tomatoes. *Cf-10*-gene-carrying line showed remarkable resistance to *Cladosporium fulvum*. To exploit its defense response mechanism, we performed a multiple-omics profiling of *Cf-10*-gene-carrying line and a susceptible line without carrying any resistance genes at non-inoculation and 3 days post-inoculation (dpi) of *C. fulvum*. We detected 54 differentially expressed miRNAs (DE-miRNAs) between the non-inoculation and 3 dpi in the *Cf-10-*gene-carrying line, which potentially regulated plant-pathogen interaction pathways and hormone signaling pathways. We also revealed 3,016 differentially expressed genes (DEGs) between the non-inoculated and 3 dpi in the *Cf-10*-gene-carrying line whose functions enriched in pathways that were potentially regulated by the DE-miRNAs. Integrating DE-miRNAs, gene expression and plant-hormone metabolites indicated a regulation network where the downregulation of miRNAs at 3 dpi activated crucial resistance genes to trigger host hypersensitive cell death, improved hormone levels and upregulated the receptors/critical responsive transcription factors (TFs) of plant hormones, to shape immunity to the pathogen. Notably, our transcriptome, miRNA and hormone metabolites profiling and qPCR analysis suggested that that the downregulation of miR9472 potentially upregulated the expression of *SAR Deficient 1* (SARD1), a key regulator for ICS1 (Isochorismate Synthase 1) induction and salicylic acid (SA) synthesis, to improve the level of SA in the *Cf-10*-gene-carrying line. Our results exploited potential regulatory network and new pathways underlying the resistance to *C. fulvum* in *Cf-10*-gene-carrying line, providing a more comprehensive genetic circuit and valuable gene targets for modulating resistance to the virus.

## 1 Introduction

Tomato (*Solanum lycopersicum*) leaf mold, which is caused by the biotrophic fungus *C. fulvum* (*Cladosporium fulvum*) (Joosten and De Wit, 1999), is a serious disease that has produced tremendous yield losses in tomato cultivation worldwide. In general, this fungus may lead to a reduction in fruit yield and quality by infecting foliage, petioles and even stems (Thomma et al., 2005). The interaction between *C. fulvum* and tomato plants has promoted the evolution of the fungus to different physiological races, and each of them has race-specific elicitor proteins that are encoded by so-called *Avr* genes (Van den Ackerveken et al., 1993). These race-specific effector proteins could be secreted into the apoplastic space during infection, which may result in compatible or incompatible interactions between *C. fulvum* and the host plant, where compatible interactions can occur if the plant is able to prevent the infection (Hammond-Kosack and Jones, 1996; De Wit, 2016). *Cf* genes are resistance genes (R-genes) that encode extracellular membrane-anchored glycoproteins comprised mainly of 24 amino acid leucine-rich repeats that mediate the recognition of Avr proteins and trigger defense responses, such as hypersensitive responses, to restrict pathogen growth (Thomas et al., 1998; Haanstra et al., 2000). Arm races between *C. fulvum* and tomato plants have driven the fungus to evolve new Avr variations that escape from the recognition of host *Cf* genes (Joosten and De Wit, 1999; Maor and Shirasu, 2005). Therefore, a genetic understanding of how *Cf* genes mediate host defense gene expression and physiological changes to acquire immunity to the fungus would help us improve host resistance.

Transcriptome analysis has been frequently employed for dissecting the genetic mechanism underlying the defense response mediated by *Cf*-genes, such as *Cf-10*, *Cf-12*, *Cf-16* and *Cf-19* (Xue et al., 2016; Zhao et al., 2016; Liu et al., 2018; Zhang et al., 2020). Comparing the transcriptome before inoculation, 4 dpi and 8 dpi of the *Cf-12*-gene-carrying line revealed several thousands of differentially expressed genes (DEGs) whose functions were enriched in defense-signaling pathways, such as the calcium-dependent protein kinase pathway and the jasmonic acid (JA) signaling pathway (Xue et al., 2016), suggesting that the *Cf-12* gene mediated the defense response by activating these pathways. Similarly, transcriptome profiling of the *Cf-16*-gene-carrying line and susceptible line, which did not include any resistance genes before inoculation, at 4 dpi and 8 dpi, showed that the upregulated genes in the *Cf-16*-gene-carrying line were primarily linked to defense processes, such as salicylic acid (SA) and jasmonic acid (JA) signaling pathways and phytohormone signaling (Zhang et al., 2020). Correspondingly, the levels of SA and JA were increased at 8 dpi and 4 dpi, respectively, in the *Cf-16*-gene-carrying line, suggesting that activating SA- and JA-associated pathways are the genetic mechanism underlying the *Cf-16*-mediated defense response. Meanwhile, our transcriptome investigation of the *Cf-19-*gene*-*carrying line before inoculation and at 7 dpi and 20 dpi also showed the activation of plant-hormone signaling pathways, including the JA and SA signaling pathways, as well as improved levels of JA and SA at 7 dpi (Zhao et al., 2016). Furthermore, our other transcriptome and physiological investigations of the *Cf-10*-gene-carrying line before inoculation and 16 dpi showed that DEGs between the two time points were primarily associated with defense-signaling pathways, including oxidation-reduction processes, oxidoreductase activity and plant hormone signal transduction. The levels of super oxidase family members, including superoxide dismutase (SOD), catalase (CAT) and peroxidase (POD), were increased at 16 dpi, while JA potentially participated in the resistance response and may only occur during the initial infection period ((Liu et al., 2018). In summary, transcriptome studies of the *Cf-10*, *Cf-12*, *Cf-16* and *Cf-19* carrying line clearly showed that their defense response mechanism involved in the activation of defense response pathways and plant hormone signaling pathways, especially the JA and SA signaling pathways. However, genetic circuit which tells what components regulated these previously known important pathways for modulating defense response is still unknown.

MicroRNAs (miRNAs) have been intensively reported to play various regulatory roles at the transcriptional or posttranslational level during the plant response to pathogen infection (Staiger et al., 2013). In general, miRNAs contribute to the plant defense response by two mechanisms: forming a miRNA-induced silencing complex to silence exogenous mRNAs and activating defense response pathways (Wang and Galili, 2019). In response to fungal invasion, miRNAs have been observed to activate disease resistance genes by acting as negative or positive regulators of these genes. For instance, slmiR482f and slmiR5300 were suppressed during *Fusarium oxysporum* infection in tomato, while the expression of two predicted targets of slmiR482f and slmiR5300, the R genes *tm-2* and *Solyc08g075630*, was increased after inoculation, demonstrating that the two miRNAs play a negative role during the defense response (Ouyang et al., 2014). miRNAs have also been shown to improve disease resistance by interacting with plant hormone pathways. For instance, miR393 has been reported to be involved in the regulation of the auxin signaling pathway by activating PAMP-triggered immunity during the antibacterial response of *Arabidopsis thaliana* and other plants (Iglesias et al., 2014). In tomato, an investigation demonstrated that the miR319/TCP4 module mediated resistance to root-knot nematodes by regulating JA synthetic genes and therefore improved endogenous JA levels (Zhao et al., 2015). Given that miRNAs have widely regulated plant hormone signaling pathways and plant defense response pathways during fungal invasion and that *Cf* genes activate host immunity to *C. fulvum* by regulating the expression of plant hormones and defense response pathways, miRNAs are likely components that link *Cf* genes and downstream defense-associated pathways.

To this end, we attempted to unveil the defense response mechanism of the *Cf-10*-gene-carrying line by profiling miRNAs, transcriptome and plant hormones and their metabolites at non-inoculation and 3 dpi for both the *Cf-10*-gene-carrying line (Ontario 792) and the susceptible line without carrying any *Cf* genes (Moneymaker, MM). Our results showed that the defense response of the *Cf-10*-gene-carrying line is widely involved in the altered expression of plant hormone signaling pathways and plant-pathogen interaction pathways and the downregulation of miRNAs whose potential target genes are critical defense response genes and plant hormone receptors/responsive transcription factors. Simultaneously, integrating differentially expressed miRNAs, gene expression patterns and plant hormones and their metabolites further suggested that a miRNA-guided “trade-off” between growth and disease resistance is the potential genetic mechanism underlying the defense response to *C. fulvum* infection in *Cf-10*-gene-carrying line.

## 2 Materials and methods

### 2.1 Plant materials

Two tomato lines, Ontario 792 which carried *Cf-10* gene (hereafter *Cf-10-*gene-carrying line) and Moneymaker which without carried any Cf genes (hereafter *C. fulvum* susceptible line), which were donated by the Institute of Vegetables and Flowers, Chinese Academy of Agricultural Sciences, were used as the materials for this study. The culture of seedlings, sporangia inoculation, staining and microscopic observation experiments were performed as described in our previously published paper (Liu et al., 2018). Briefly, a suspension of *C. fulvum* (physiological Race 1.2.3.4 at 1 × 107 sporangia per mL) was used to inoculate the abaxial leaf surfaces of 30 plants per line at the 4-6 leaf stage. Approximately 0.1% 3,3′-diaminobenzidine (DAB) and 0.2% nitrotetrazolium blue chloride (NBT) were used to detect the accumulation of H_2_O_2_ and O^2−^ in plant leaves, respectively (Rao and Davis, 1999; Kumar et al., 2014). A Quanta 200FEG microscope (Field Electron and Ion, United States) and an OLYMPUS SZX10 microscope (Olympus, Japan) were used for microscopic observation. Young leaves at 0 days post-inoculation (dpi) and 3 dpi were harvested and placed in liquid nitrogen for total RNA isolation and hormone determination with three replications.

### 2.2 Total RNA extraction

Total RNA of leaves with three biological repetitions was isolated from the non-inoculated and inoculated plants of susceptible line and *Cf-10*-gene-carrying line using TRIzol^®^ reagent (Invitrogen, Carlsbad, CA, United States) by following the manufacturer’s instructions. Approximately 0.5 g of leaves from the same plant were collected, and total RNA was extracted from the mixed sample. The quality of the RNA, including concentration and purity, was evaluated by an optical device. The high-quality RNA samples were then stored at −80°C for further library construction and qRT–PCR.

### 2.3 Library construction and sequencing

After quality control of total RNA samples, sequencing libraries were constructed. RNA-seq libraries were prepared according to Xue’s method (Xue et al., 2016). Briefly, 1) mRNA molecules were enriched from the total RNA samples by using oligo-dT attached magnetic beads; 2) mRNA molecules were broken into small random fragments by using a fragmentation buffer; and 3) first-strand cDNA was synthesized by using random hexamers, whereas second-strand cDNA was synthesized by using DNA polymerase I, RNase H and dNTPs. Then, synthesized cDNAs were purified by AMPure XP beads. 4) End repair was performed on the cDNA fragments by the addition of a single “A” base and ligated adapters. AMPure XP beads were added to select cDNA with an appropriate length for the PCR enrichment process to finalize the library preparation process. Sequencing of the quality control (QC)-passed libraries was performed on an NGS Illumina NovaSeq platform with a paired-end (PE) 2 × 150 bp model. For miRNA library construction, small RNAs were precipitated by polyethylene glycol and separated by 15% denaturing PAGE gel purification (16–28 nt). Small RNA libraries were constructed using TruSeq Small RNA Sample Prep Kits (Illumina, San Diego, USA) in accordance with the manufacturer’s protocol, and single-end sequencing was performed on an Illumina HiSeq 2,500 sequencer. All the clean sequencing data were uploaded to the Sequence Read Archive (SRA) database of NCBI under accession number PRJNA781749.

### 2.4 Bioinformatic analysis of miRNAs

Raw miRNA sequencing reads were carried out as follows: 1) adaptors were removed by using Cutadapt (Martin, 2011); 2) reads longer than 30 nt or shorter than 18 nt were removed; and 3) low-quality reads with a missing rate >10% were removed for each sample. To this end, we obtained 9.68 M high-quality reads. To detect the expression of known miRNAs, we first mapped the high-quality reads against the reference genome of Solanum lycopersicum (ITAG4.0) by SOAP2 (Li H et al., 2009) and then mapped the reads against the known miRNAs from miRBase (V22) using the following parameters: 1) validated region of reference miRNAs: 2 nt and +5 nt of the miRNAs; 2) tolerating one mismatch.

For reads that could be mapped to the reference genome but were unable to be mapped to known miRNAs, we employed miRDeep2 (Friedländer et al., 2012) to predict their potential as novel miRNAs by a Bayes-based scoring system that summarized the common features of miRNAs: potential mature, star and loop structure and predicted the secondary structures of potential precursors (predicted by the RNAfold randfold function of miRDeep2). Given that miRNAs belonging to the same family are highly conserved during evolution, we clustered these novel miRNAs with known miRNAs from miRBase to classify the families of these novel miRNAs.

We then employed TargetFinder (Xie K et al., 2012) to predict the potential target genes of the known and novel miRNAs, by using the sequence of genes assembled from transcript as the reference database and BLAST the miRNAs against the database for searching potential target genes. miRNA-gene pairs which had a score of higher than 1.5 were selected as the candidates in the study. The functions of these putative target genes were predicted by BLAST against NR (Pruitt et al., 2005), Swiss-Prot (Apweiler et al., 2004), GO (Ashburner et al., 2000), COG (Tatusov et al., 2000), KEGG (Kanehisa et al., 2004), KOG (Koonin et al., 2004) and Pfam (Eddy, 1998). Enrichment of the putative genes on GO modules/KEGG pathways was tested with Fisher’s extract test.

To exploit differentially expressed miRNAs before and after inoculation of *C. fulvum* in both the *Cf-10*-gene-carrying line and susceptible line and between the resistant and susceptible lines after inoculation, we quantified the expression of miRNAs with the Trans Per Kilobase of exon model per million mapped reads (TPM) (Wagner et al., 2012) and exploited differentially expressed miRNAs by using the R package edgeR (Karasov et al., 2017) and |Fold-change| ≥ 1.5 and *p*-value <0.05 as the cutoff of significance.

### 2.5 Transcriptome sequencing data analysis

Quality control of raw sequence data for all samples was conducted with a customized script as follows: 1) adaptors were removed and 2) low-quality reads (reads with proportion of N > 10% or with >50% of bases with a low Phred quality score (Q ≤ 10) were removed. The clean reads were aligned against the tomato reference genome (Solanum lycopersicum ITAG4.0) using HISAT2 (Kim et al., 2019). The mapped reads were processed with Samtools (Li R Q et al., 2009). Transcript assembly was then performed for each sample individually with StringTie (Pertea et al., 2015) with the mapped reads and gene annotation of the reference genome. The assembled transcripts were merged as a new reference and employed as a reference during quantification of the expression of every sample.

Four comparisons were conducted for exploiting differentially expressed genes (DEGs): *Cf-10*-gene-carrying line 3 dpi vs. *Cf-10*-gene-carrying line before inoculation, susceptible line 3dpi vs. susceptible line before inoculation and Cf-10-gene-carrying line 3dpi vs. susceptible line after inoculation. DEG analysis was carried out for each comparison with DESeq2 (Love et al., 2014) with cutoffs of |Fold Change| ≥ 1.5 and *p*-value <0.05 to define statistical significance. Similar to the functional annotation of miRNA target genes described above, the functions of DEGs were annotated by BLAST to NR, Swiss-Prot, GO, COG, KEGG, KOG and Pfam. Enrichment of the GO module and pathway of the DEGs was tested with Fisher’s extract test.

Transcript factors were annotated by following the PlantTFDB database (Jin et al., 2016). Transcript factors and miRNAs which deferentially expressed in any of the comparisons were employed as the input. Then the transcript factor-transcript factor co-expression network and transcript factor-miRNA co-expression network were constructed by using R package WGCNA (Langfelder and Horvath, 2008). A WGCNA soft threshold power of 11 was employed as a cutoff during the co-expression similarity and adjacency calculation step.

### 2.6 qRT–PCR

The total RNA of Ontario 792 and Moneymaker at 0 dpi and 3 dpi was selected for qRT-PCR. The primers for candidate genes were designed using Primer 5.0 software (Supplementary Table S15). Actin-EFα1 was used for internal normalization. qRT-qPCR was performed in an iQ5 system (Bio-Rad, United States). The relative gene expression levels were calculated using the 2^−ΔΔCT^ method (Livak and Schmittgen, 2001).

### 2.7 LC-MS/MS analysis

The leaf samples (50 mg) were ground into powder, and then 10 μL (100 ng/mL) of internal standard mixed solution and 1 mL of methanol/water/formic acid (15:4:1, v/v/v) extracting solution were added gradually with 10 min of vortex mixing. The mixed liquor was centrifuged at 4°C at 12,000 r/min, and the supernatant was taken into a new centrifuge tube for concentration, followed by 100 μL of 80% methanol/water solution for dissolution and 0.22-μm filtering. The final extracting solution was placed in an injection bottle for LC-MS/MS analysis. UPLC conditions (Applied Biosystems 6,500 Triple Quadrupole) and ESI-MS/MS conditions (QTRAP^®^ 6,500+ LC-MS/MS System) were used for LC-MS/MS analysis according to previously published papers (Niu et al., 2014; Šimura et al., 2018). All samples were qualitatively and quantitatively analyzed using Analyst 1.6.3 (Sciex, Darmstadt, Germany) and Multiquant 3.0.3 (Sciex, Darmstadt, Germany). Significantly regulated metabolites between groups were determined by *t*-test for the *p*-value (*p* ≤ 0.1) and absolute Log2FC (|Fold Change| ≥ 1.5). The identified metabolites were annotated using KEGG (Kanehisa et al., 2004).

## 3 Results

### 3.1 Different performance to the infection of *C. fulvum* between *Cf-10* gene carrying and susceptible lines

**FIGURE 1:**
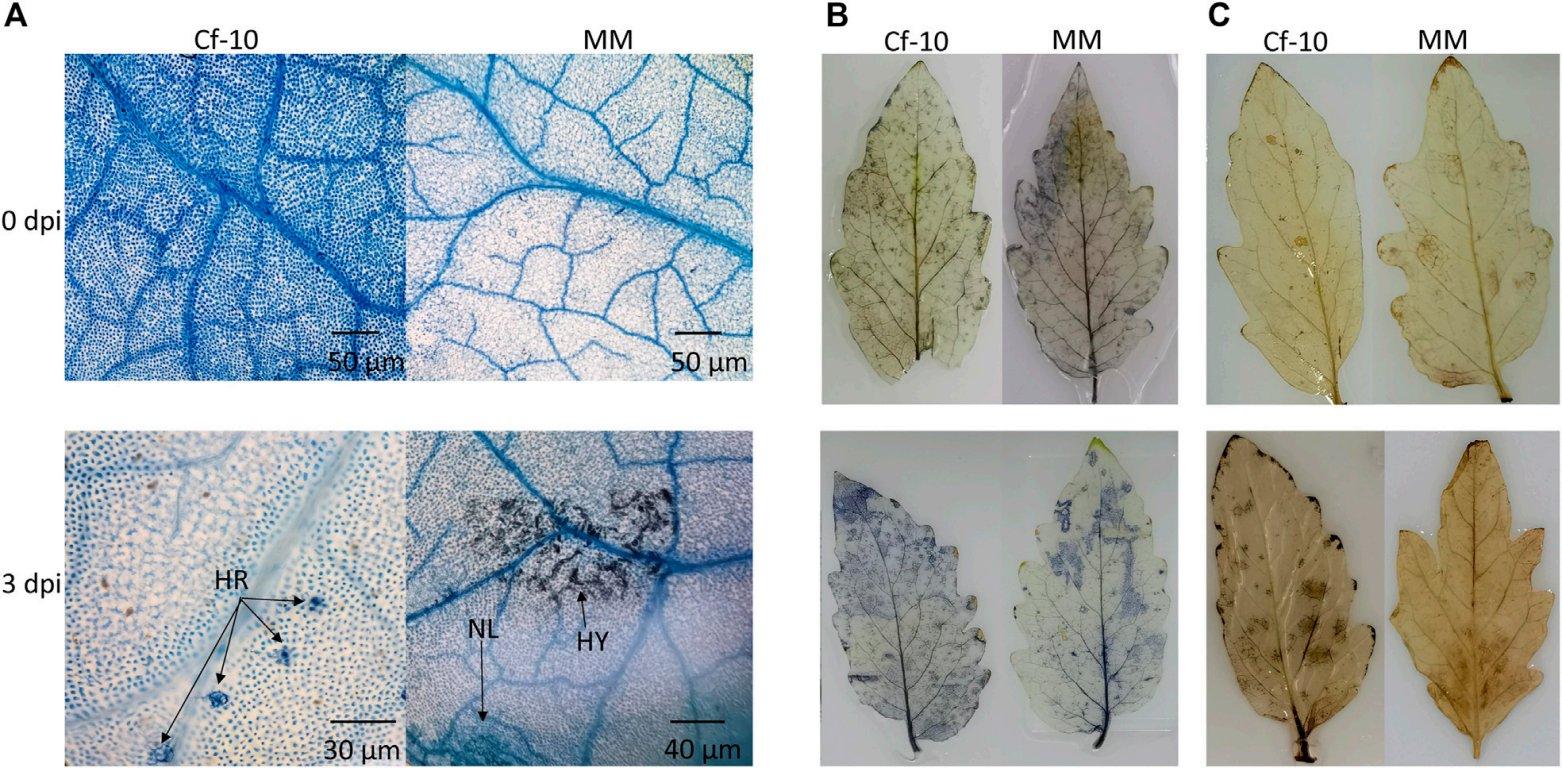
*Cf-10* gene carrying and susceptible lines have different response patterns to the invasion of *C. fulvum* at 3 days post-inoculation (dpi). **(A)**. Observable hyphal germination and cell death in the susceptible line at 3 dpi (bottom left) compared with that of the *Cf-10*-gene-carrying line (bottom right) **(B–C)**. The *Cf-10*-gene-carrying line accumulated more superoxide (B: bottom left) and hydrogen peroxide (C: bottom left) than the susceptible line (bottom right of B and C) at 3 dpi. The blue color in **(B)** and brown color in **(C)** on the leaves indicate the levels of superoxide and hydrogen peroxide, respectively. HR: hypersensitive response; HY: hypha; NL: necrotic lesion.

Inoculating the *Cf-10*-gene-carrying line (Ontario 792) and susceptible (Moneymaker, MM) line with 10 most prevalent physiologic races of *C. fulvum* demonstrated that the former showed resistance to 8 races and the latter had no resistance to any of the races (Liu et al., 2018), indicating *Cf-10*-gene-carrying line has wide spectrum of resistance to the virus. To this end, we inoculated both lines with 1.2.3.4 physiologic races which could invade to the susceptible line but was unable to infect *Cf-10*-gene-carrying line to study defense mechanism underlying *Cf-10*-gene-carrying line. Expression profiling showed that the expression level of the candidate gene of *Cf-10* locus inferred by our previous study (Liu et al., 2018) was peaking at 3 dpi (Supplementary Figure S1A).Trypan blue staining was used to monitor the infection progress and response. The scanning electron and optical microscopy results displayed an obvious difference between the resistant and susceptible lines at 3 dpi in the hypersensitive response (HR) areas in the former and fungal spots in the latter (Figure 1A). Moreover, the susceptible line showed more visible disease spots in leaves than the *Cf-10*-gene-carrying line at 10 dpi (Supplementary Figure S1). Investigating the cell death and superoxide and hydrogen peroxide levels in both lines at 6 h, 1, 2, 3, 4, 5, 8, and 10 days after inoculation indicated that resistant and susceptible line show phenotypic difference only at 3 days after inoculation and the following time points (Supplementary Figure S1B and Supplementary Figure S1C), where the ROS accumulation status indicated a relatively higher signal of both H_2_O_2_ and O^−2^ in the *Cf-10*-gene-carrying line than in the susceptible line at 3 dpi (Figures 1B,C), indicating that ROS played a role in plant defense against pathogens (PTI and ETI). These results indicated that the two lines showed distinct responses after *C. fulvum* infection at early stages.

### 3.2 miRNA sequencing of *Cf-10*-gene-carrying line and susceptible line for the non-inculation and 3 dpi of *C. fulvum*

To reveal miRNAs responding to the inoculation of *C. fulvum*, we conducted genome-wide expression profiling of miRNAs in susceptible and *Cf-10*-gene-carrying lines. In total, we detected 138 known miRNAs, with numbers ranging from 108 to 127 across samples (Supplementary Table S1). Furthermore, we predicted novel miRNAs in the context of their biological properties, such as hair-pin structure, using miRDeep2 (Friedländer et al., 2012). We identified 310 novel miRNAs in total, with numbers ranging from 308 to 310 across samples (Supplementary Table S1). These miRNAs belong to 107 miRNA families (Supplementary Table S2), suggesting a wide occurrence of miRNA-mediated gene regulation during the invasion of the fungus.

We then predicted the potential target genes of these miRNAs by using TargetFinder (Xie F L et al., 2012). In total, we revealed 3,233 potential target genes for 375 miRNAs, including 2,226 genes for 129 known miRNAs and 1,166 for 246 novel miRNAs. The number of miRNA target genes ranged from 2,885 to 3,166 across samples. The functions of the 3,132 predicted target genes (99.99% of the total predicted target genes) were annotated by using several gene functional databases, including NR (Pruitt et al., 2005), Swiss-Prot (Apweiler et al., 2004), GO, COG (Tatusov et al., 2000), KEGG (Kanehisa et al., 2004), KOG (Koonin et al., 2004) and Pfam (Eddy, 1998).

### 3.3 MicroRNA expression profiling of tomato under the inoculation of *C. fulvum*

**FIGURE 2:**
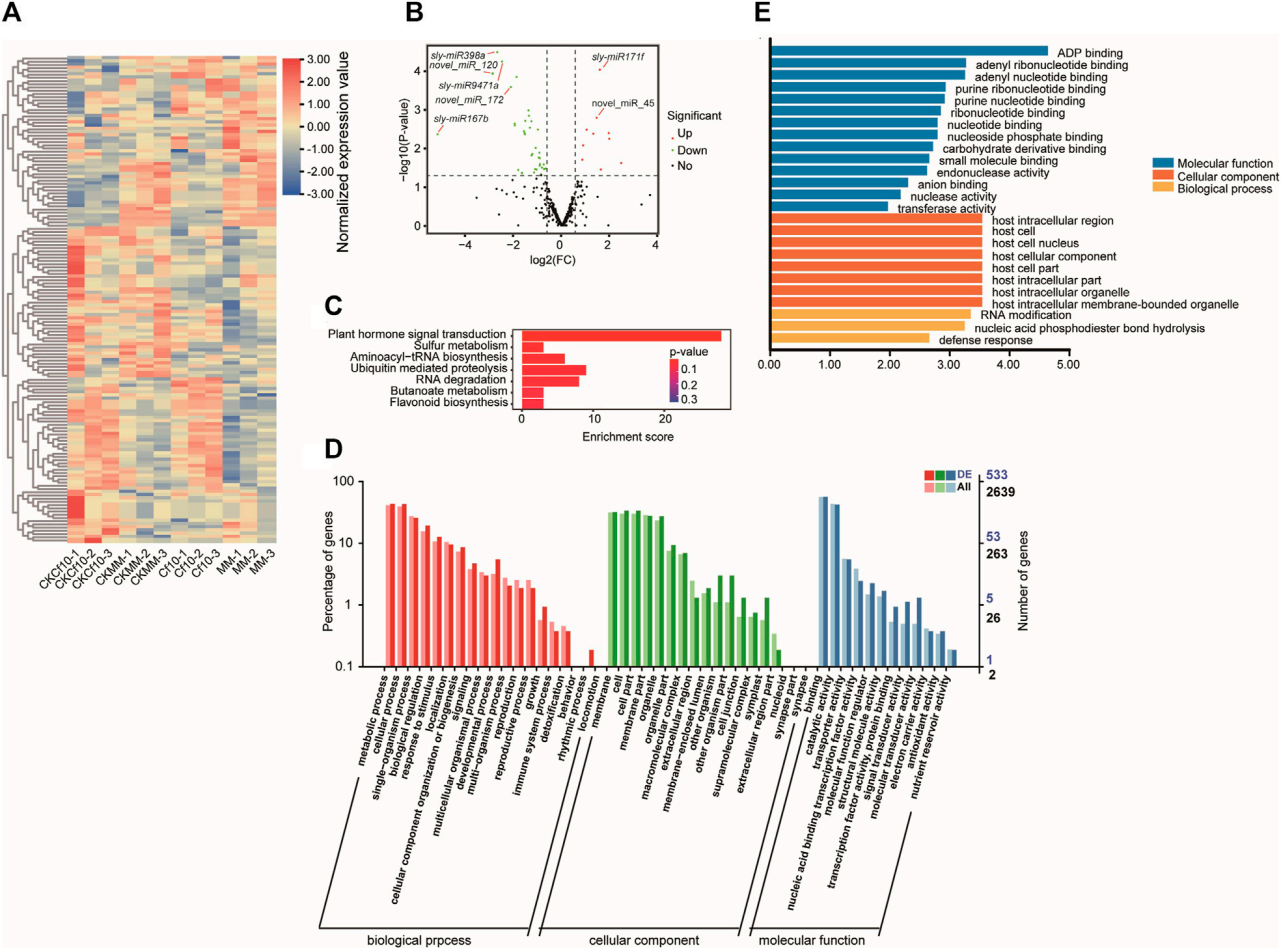
miRNA profiling of *Cf-10*-gene-carrying lines carrying the *Cf-10* gene for the non-inoculation and 3 dpi of *C. fulvum*. **(A)** Expressional patterns of differentially expressed miRNAs between the control and 3 dpi *Cf-10-*gene-carrying lines across samples. CKCf10: *Cf-10*-gene-carrying line under control conditions; CKMM: susceptible line under non-inoculation condition; Cf10: *Cf-10*-gene-carrying line at 3 dpi; MM: susceptible line at 3 dpi. **(B)** Volcano plot of differentially expressed miRNAs between the control and 3 dpi *Cf-10*-gene-carrying lines. Red dots are upregulated, while green dots are downregulated genes at 3 dpi in the *Cf-10*-gene-carrying line. Black points are insignificant genes. Name of miRNAs were marked on the plot. **(C)** KEGG pathway and GO **(D)** enrichment analysis of predicted target genes of differentially expressed miRNAs between the control and 3 dpi *Cf-10*-gene-carrying lines. **(E)**. GO enrichment analysis of predicted target genes of downregulated miRNAs.

We quantified the expression of miRNAs for the control and 3 dpi of *C. fulvum* and exploited differentially expressed miRNAs (DE-miRNAs) by calculating the log2Ratio and using DESeq2 (Love et al., 2014). By using |Fold-change| ≥1.5 and DESeq2 *p*-value <0.05 as cutoffs, we identified 54 differentially expressed miRNAs between the non-inocluation and 3 dpi in *Cf-10*-gene-carrying lines, including 43 downregulated and 11 upregulated at 3 dpi (Figures 2A,B and Supplementary Table S3). We also detected 55 differentially expressed miRNAs between the non-inocluation and 3 dpi in susceptible lines, including 28 downregulated and 27 upregulated miRNAs at 3 dpi (Supplementary Table S4 and Supplementary Figure S2A). Comparing the two sets indicated 13 shared and 42 *Cf-10* gene-carrying line-specific differentially expressed miRNAs. All 13 shared differentially expressed miRNAs were downregulated at 3 dpi, except sly-miR9472-5p, which was downregulated in the *Cf-10*-gene-carrying line but upregulated in the susceptible line. In addition, comparing miRNAs between the resistant and susceptible lines at 3 dpi further revealed 68 differentially expressed miRNAs, including 30 downregulated and 38 upregulated miRNAs in the *Cf-10*-gene-carrying line (Supplementary Table S5). Furthermore, comparing *Cf-10*-gene-carrying line and susceptible line revealed 68 differentially expressed miRNAs at 3 dpi, 30 of which were upregulated in the *Cf-10*-gene-carrying line, but 38 of which were downregulated in the *Cf-10*-gene carrying line at 3 dpi. In summary, our data suggested that the downregulation of miRNAs in the *Cf-10*-gene-carrying line was widespread after the invasion of *C. fulvum*, possibly indicating that miRNAs play negative roles in the defense response to the fungus. Compared to the susceptible line, the *Cf-10*-gene-carrying line specifically downregulated many miRNAs after the invasion of the fungus.

### 3.4 Functional enrichment of the predicted target genes of the differentially expressed miRNAs

To interpret the biological functions of the differentially expressed miRNAs, we conducted functional enrichment analysis of their predicted target genes with the convenience of gene functional annotation from the GO and KEGG databases (Ashburner et al., 2000; Kanehisa et al., 2004). For the *Cf-10*-gene-carrying line, we annotated the function for 894 target genes of the 54 differentially expressed miRNAs between the non-inoculation and 3 dpi (Supplementary Table S6). KEGG enrichment analysis also indicated that significantly enriched pathways (*p*-value<0.05) were “plant hormone signal transduction”, “sulfur metabolism,” “aminoacyl-tRNA biosynthesis,” “aminoacyl-tRNA biosynthesis” and “RNA degradation” (Figure 2C). GO enrichment analysis revealed that the top enriched biological terms were “transcription,” “gene silencing by RNA,”, “development process,” “response to auxin” and “cellular response to DNA damage stimulus”. The top enriched molecular function terms were “ubiquitin protein ligase binding,” “sulfate adenylyltransferase (ATP) activity” and “O-methyltransferase activity.” The top enriched cellular component terms were “host cell nucleus,” “organelle membrane” and “clathrin-coated vesicle” (Figure 2D), all of which are associated with the host defense response. GO enrichment analysis for the predicted target genes of downregulated miRNAs showed that they enriched in the terms involved in host cellular component and defense response (Figure 2E). Notably, JASMONIC ACID-AMIDO SYNTHETASE (JAR1) (Staswick and Tiryaki, 2004), a critical gene for JA signaling and involved in the crosstalk between JA and auxin, was predicted to be the target gene of miR156 (Supplementary Table S6). In addition to these overrepresented pathways, we also observed 38 genes from plant–pathogen interaction pathways, and 3 genes that regulated apoptotic processes and programmed cell death were also potential targets of the putative miRNAs (Supplementary Table S6). Therefore, altered expression of miRNAs to activate defense response genes might be the genetic mechanism underlying the strong resistance to *C. fulvum* infection in the *Cf-10*-gene-carrying line.

For the susceptible line (Supplementary Table S7 and Supplementary Figure S2), the top enriched GO terms of the predicted target genes of the differentially expressed miRNAs were “lignin catabolic process,” “regulation of shoot system development,” “defense response to fungus,” “response to salicylic acid,” “tricarboxylic acid cycle,” “apoplast,” “hydroquinone:oxygen oxidoreductase activity” and “copper ion binding” (Supplementary Figure S2B), while the top enriched KEGG pathways were “stilbenoid, diarylheptanoid and gingerol biosynthesis,” “ether lipid metabolism” and “porphyrin and chlorophyll metabolism” (Supplementary Figure S2C). For target genes of differentially expressed miRNAs between resistant and susceptible lines at 3 dpi, the top enriched GO terms were those associated with defense responses, such as “defense response,” “response to abiotic stimulus” and “positive regulation of abscisic acid-activated signaling pathway,” and the top enriched KEGG pathways were “plant–pathogen interaction,” “plant hormone signal transduction” and “ubiquitin mediated proteolysis”, suggesting that the *Cf-10*-gene-carrying line had different genetic regulation of defense response pathways when compared to that of the susceptible line. The difference in resistance between resistant and susceptible lines might result from the different expression patterns of miRNAs between the two lines.

### 3.5 Transcriptome profiling identified defense response genes and pathways

**FIGURE 3:**
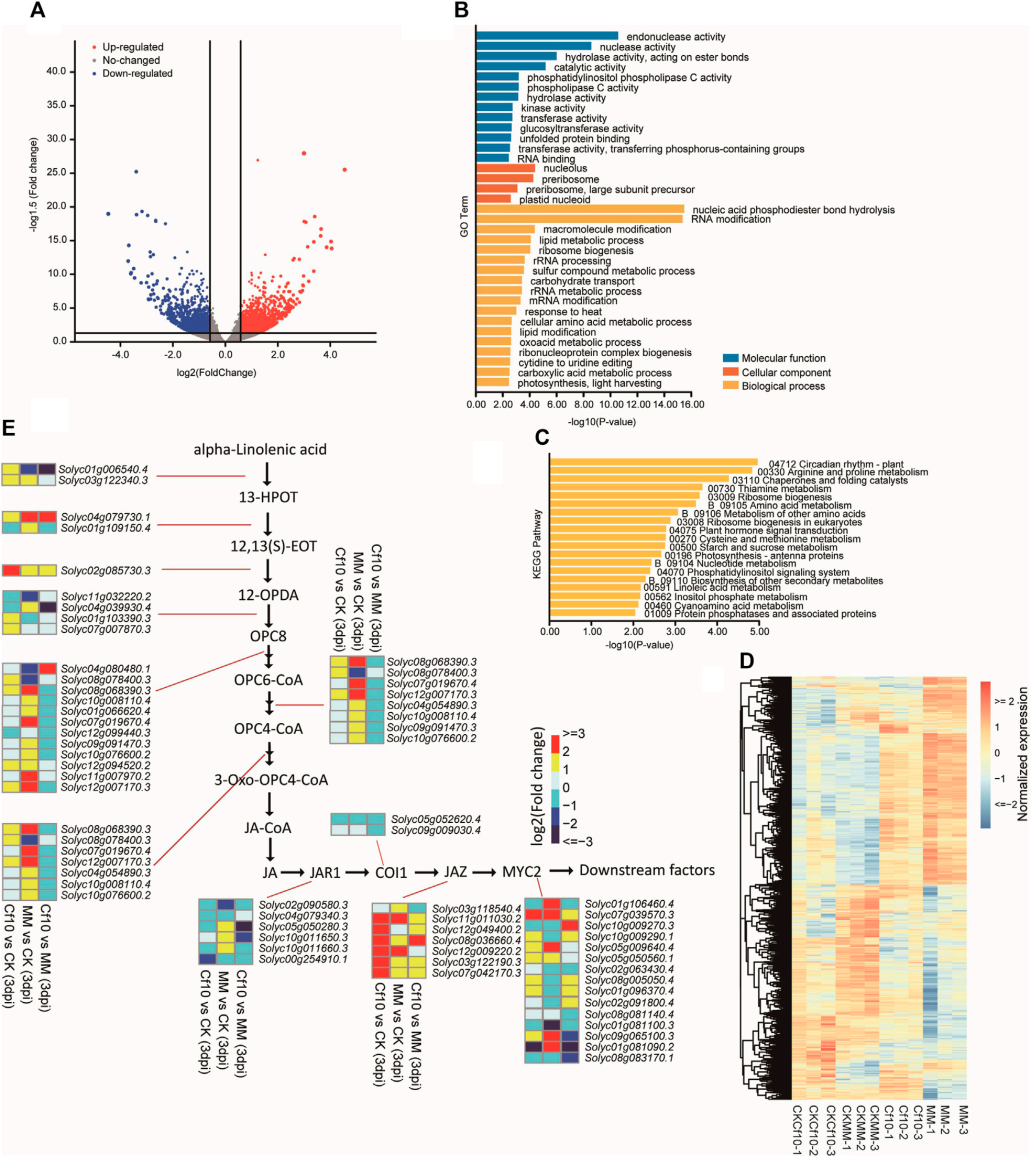
Transcriptome profiling revealed differentially expressed genes between the non-inoculation and 3 dpi *Cf-10*-genecarrying line that carries the *Cf-10* gene. **(A)** Volcano plot comparing gene expression levels between the control and 3 dpi *Cf-10*-gene-carrying line. Red dots are upregulated, while green dots are downregulated genes in the 3 dpi *Cf-10*-gene-carrying line. Black points are insignificant genes. **(B)** GO and KEGG pathway **(C)** enrichment analysis of differentially expressed genes between the non-inoculation and 3 dpi *Cf-10*-gene-carrying line. **(D)** Heatmap of DEGs. **(E)** Expression pattern of genes involved in JA synthesis and signaling pathways.

To exploit the genes responding to the invasion of the fungus, we conducted transcriptome profiling of the leaves of both resistant and susceptible lines for both the control and 3 dpi. We identified 3,016 DEGs with the criteria of |Fold Change (FC)| ≥ 1.5 and *p*-value < 0.05 to compare the control and 3 dpi *Cf-10*-gene-carrying lines (Figures 3A, D and Supplementary Figure S4A), with 1,658 upregulated and 1,358 downregulated genes (Supplementary Table S8). Similar to our previous study for 16 dpi (Liu et al., 2018), we observed DEGs between the non-inoculation and 3 dpi *Cf-10*-gene-carrying lines enriched in canonical pathogen response pathways, including the “oxoacid metabolic process,” “circadian rhythm,” “arginine and proline metabolism,” and “regulation of jasmonic acid mediated signaling pathway” (Figures 3B,C and Supplementary Table S8), a large proportion of which had at least one gene belonging to the potential targets of DE-miRNAs between the control and 3 dpi in the *Cf-10*-gene-carrying line. We also observed that almost every step of JA synthesis and signaling pathways was differentially expressed between the control and 3 dpi in the *Cf-10*-gene-carrying line (Figure 3E), where genes from JA synthesis and signaling pathways were generally upregulated at 3 dpi, especially genes from JA signaling pathways, such as *JAZ* and *MYC2*.

DEGs between the resistant and susceptible lines (Supplementary Table S10 and Supplementary Figure S4A) at 3 dpi were enriched in defense response- and growth-associated GO terms, such as “photosynthesis,” “oxidoreductase activity,” “defense response to bacterium”, “plastid membrane” and “proline metabolic process” (Supplementary Figure S4B and Supplementary Table S9). Meanwhile, these DEGs were enriched in KEGG pathways that were also associated with the defense response and plant growth, including “photosynthesis-antenna proteins”, “Energy metabolism”, and “circadian rhythm” (Supplementary Figure S4C). These results further indicated that the *Cf-10*-gene-carrying line had different genetic regulation of defense response pathways when compared to that of the susceptible line. Different expression patterns of growth-related genes might also result from the different genetic regulation of defense response pathways between the two lines.

Comparison of non-inoculation and 3 dpi in susceptible line identified 8,106 DEGs (Supplementary Figure S4D and Supplementary Table S9). GO and KEGG enrichment analysis showed that the DEGs enriched in gene translation processes, e.g., “ribosome biogenesis”, “translation” and “RNA processing” (Supplementary Figures S4E, F). Comparing DEGs among different comparisons found 510 genes were detected by all of the comparison, 1,354 genes were detected by the comparison of non-inoculation and 3 dpi at *Cf-10*-gene-carrying line and the comparison of non-inoculation and 3 dpi at susceptible line, and 1854 genes were detected by the comparison of non-inoculation and 3 dpi at susceptible line and the comparison of *Cf-10*-gene-carrying line and susceptible line at 3 dpi (Supplementary Figure S4G), further suggesting that *Cf-10*-gene-carrying line had its specific DEGs which might enhance its disease resistant ability.

### 3.6 Integrating putative miRNA and the expression of predicted target gene informs genetic regulatory mechanisms

Integrating putative miRNAs and the transcriptome data suggested 41 differentially expressed miRNAs between the non-inoculation and 3 dpi *Cf-10*-gene-carrying line, which were predicted to regulate 183 DEGs in that comparison (Supplementary Table S11), which formed 264 potential miRNA-gene pairs. We observed that the expression of miRNAs negatively correlated with their potential target genes in 161 of the predicted miRNA-gene pairs. Meanwhile, 46 of the predicted miRNA-gene pairs showed that both putative miRNAs and their predicted target genes were upregulated, and 57 of these pairs showed both of which were downregulated at 3 dpi, which might result from the more complicated situation where the predicted target genes were regulated by miRNAs and other factors like transcript factors or epigenetic modifications.

**FIGURE 4:**
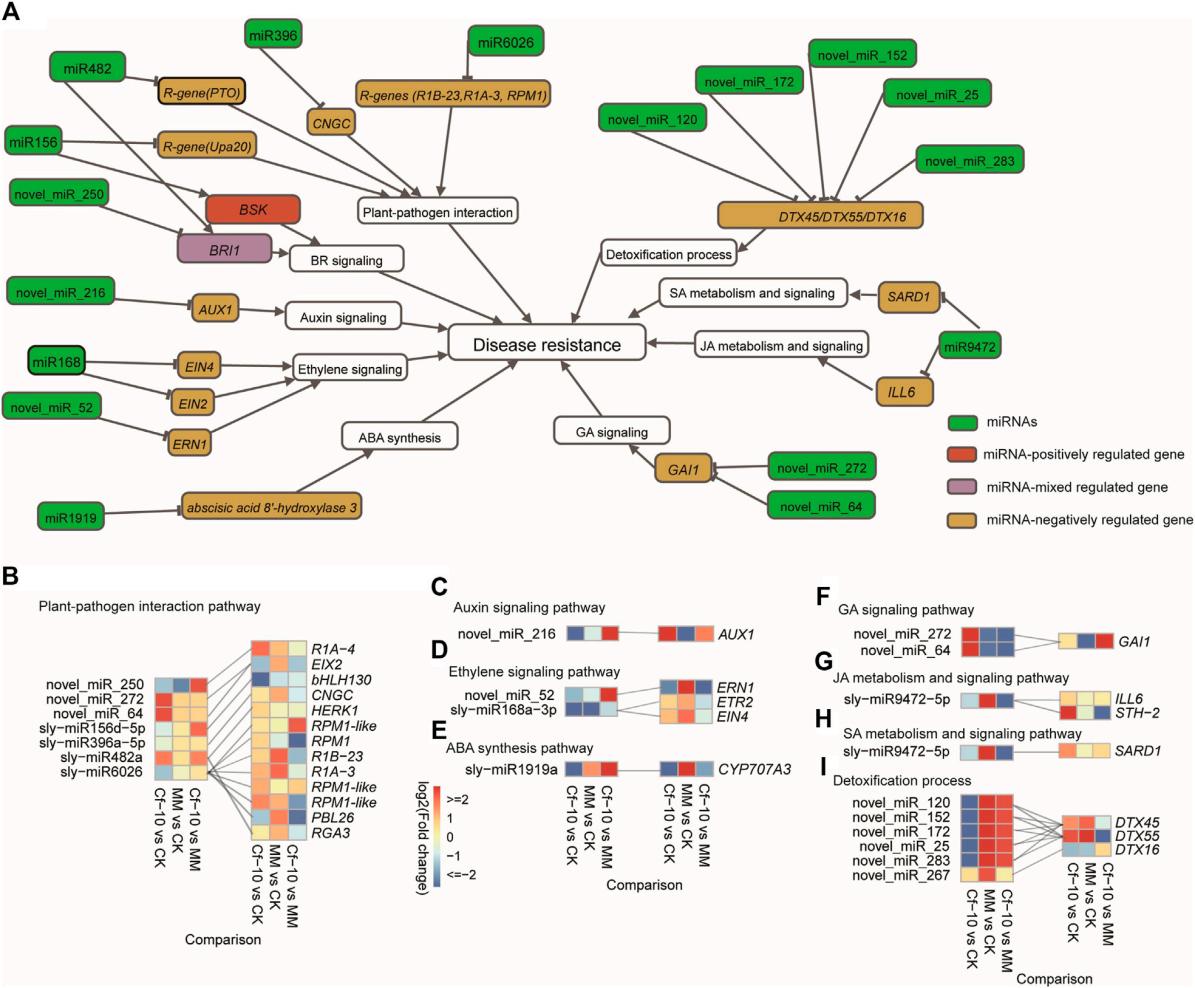
Integrating differentially expressed miRNAs and gene expression patterns demonstrated the genetic regulatory mechanism underlying disease resistance conferred by the *Cf-10* gene. **(A)** Landscape of the genetic regulation of miRNAs on disease resistance in a *Cf-10*-gene-carrying line that carries the *Cf-10* gene. **(B–H)** miRNAs potentially negatively regulated plant-pathogen interaction pathways **(B)**, the auxin signaling pathway **(C)**, ethylene signaling pathway **(D)**, ABA synthesis **(E)**, GA signaling pathway **(F)**, JA metabolism and signaling pathway **(G)**, SA metabolism and signaling pathway **(H)** and detoxification process **(I)** to confer disease resistance to *C. fulvum* in a *Cf-10-*gene*-*carrying line that carries the *Cf-10* gene. Heatmaps show miRNA (left)/gene (right) expression patterns in susceptible and *Cf-10*-gene-carrying lines for the control and 3 dpi. Gray lines between miRNAs and genes suggest the potential regulation from the former to the latter.

Investigating the function of the genes from the 263 predicted miRNA-gene pairs indicated that miRNAs potentially regulated widespread of crucial avirulence gene recognition genes and signal transduction genes in the plant-pathogen interaction pathway and the receptors/key response transcription factors (TFs) of plant hormone signaling pathways (Figure 4A). For plant–pathogen interaction pathways, miR6026 was predicted to regulate three important avirulence recognition genes (Figures 4A,B), late blight resistance protein homologs R1B-23 and R1A-3 and resistance to *P. SYRINGAE* PV MACULICOLA 1 (RPM1) (Grant et al., 1995), with miR6026 downregulated and the three genes upregulated in the 3 dpi *Cf-10*-gene-carrying line; miR482 was predicted to regulate avirulence gene recognition genes PTO (Martin et al., 1993), where both were upregulated in the 3 dpi *Cf-10*-gene-carrying line. miR156 was predicted to regulate avirulence gene master regulator Upa20 (Kay et al., 2007), with both downregulated; miR396 regulated calcium ion gated channel gene CNGC (Moeder et al., 2011), with miR396 downregulated and the CYCLIC NUCLEOTIDE GATED CHANNEL (CNGC), which was upregulated in the 3 dpi *Cf-10*-gene-carrying line. Furthermore, we detected that detoxification genes were also potentially regulated by miRNAs (Figures 4A,H), where the detoxification genes DETOXIFICATION 45 (DTX45) and DETOXIFICATION 55 (DTX55) were simultaneously predicted to be regulated by novel_miR_120, novel_miR_152, novel_miR_172, novel_miR_25 and novel_miR_283, with all of the miRNAs downregulated and the two detoxification genes upregulated in the 3 dpi *Cf-10*-gene-carrying line (Figure 4I).

For plant hormone signaling pathways, the novel miRNA novel_miR_216 was predicted to regulate the auxin influx transporter AUXIN RESISTANT 1 (AUX1) (Bennett et al., 1996), with novel_miR_216 being downregulated and AUX1 being upregulated in the 3 dpi *Cf-10*-gene-carrying line (Figures 4A,C). For the ethylene signaling pathway, miR168 was predicted to regulate ethylene responsive genes ETHYLENE RESPONSE 2 (ETR2) (Sakai et al., 1998) and response transcription factor ETHYLENE INSENSITIVE 4 (EIN4) (Hua et al., 1998), with miR168 downregulated and ETR2 (Sakai et al., 1998) and EIN4 (Hua et al., 1998) upregulated; novel miRNA novel_miR_52 was predicted to regulate ERF Required for Nodulation1 (ERN1) (Trentmann, 2000), with both downregulated (Figures 4A,D). For BR, miR156, miR482, and novel miRNA novel_miR_250 were predicted to regulate BR receptor BR INSENSITIVE 1 (BRI1) (Nam and Li, 2002) and signaling transducer BRASSINOSTEROID-SIGNALING KINASE (BSK) (Sreeramulu et al., 2013), with novel_miR_250 downregulated, BRI1 upregulated and both miR156 and BSK downregulated (Figure 4A). For ABA, miR1919 were predicted to regulate ABA biosynthesis possibly by targeting the gene encoding abscisic acid 8′-hydroxylase 3, both of which were downregulated (Figures 4A,E). For GA, the novel miRNAs novel_miR_272 and novel_miR_64 were predicted to regulate the GA response transcription factor GIBBERELLIC ACID INSENSITIVE (GAI), with both upregulated and downregulated (Figures 4A,F). Notably, for JA, miR9472 negatively regulated ILL6 (Widemann et al., 2013), where miR9472 was downregulated and ILL6 was upregulated at 3 dpi (Figures 4A,G). For SA, miR9472 negatively regulated SARD1, where miR9472 was downregulated and SARD1 was upregulated at 3 dpi (Figures 4A,H). SARD1 has been reported to be a a key regulator for ICS1 (Isochorismate Synthase 1) induction and salicylic acid (SA) synthesis, suggesting miR9472 potentially enhanced the disease resistance ability of *Cf-10* carrying by improving SA level. Beyond these pathways, we observed that miRNAs regulated the cell cycle, iron transport, ubiquitin-mediated proteolysis and genes encoding WD40-containing proteins, where iron transporter, LOG2-LIKE UBIQUITIN LIGASE4 (LUL4, ubiquitin mediated proteolysis) and cell cycle were suppressed, and WD40 and PROTEIN INHIBITOR OF ACTIVATED STAT LIKE 2 (PIAL2, ubiquitin mediated proteolysis) were upregulated.

Comparing both miRNAs and the expression of their target genes between *Cf-10* gene carrying and susceptible lines also suggested regulatory differences in defense response genes generated by differentially expressed miRNAs (Supplementary Table S12). Compared to the *Cf-10*-gene-carrying line, the upregulation of the miR156 family in the susceptible line at 3 dpi potentially regulated 41 target genes and reduced the expression level of the JA jasmonate-amido synthetase JAR1 (Staswick and Tiryaki, 2004) to suppress the JA signaling pathway, as well as genes involved in posttranscriptional modification and cell cycle control. Meanwhile, the high expression level of sly-miR482b potentially reduced the expression level of many R genes, such as RESISTANCE TO *P. SYRINGAE* 5 (RPS5) (Warren et al., 1998) and RECOGNITION OF PERONOSPORA PARASITICA 13 (RPP13) (Rose et al., 2004). Other miRNAs that were differentially expressed between the resistant and susceptible lines included miR-159, miR-160, miR-171, miR-319 and miR-9472. They were generally upregulated in the susceptible line and then reduced the expression of genes involved in the defense response, posttranscriptional modification and plant hormone signaling pathways. We observed 10 miRNAs and 53 miRNA-gene pairs that were detected in both of the comparison between before-inoculation and 3dpi in resistant line and the comparison between before-inoculation and 3dpi in susceptible line. For all of these miRNA-gene pairs, we observed that all of the miRNAs and their putative targets have been more intensively down-/upregulated in the comparison of before-inoculation and 3dpi in resistant line than that of susceptible line, further confirming the expression of miRNAs and their targets are critical for disease resistance (Supplementary Tables S11, S12).

In addition, by constructing transcript factors-transcript factors and transcript factors-miRNA co-expression network (Supplementary Figure S5), we observed that transcript factors have 7 co-expression modules. miRNAs regulated 6 out of the 7 modules and potentially act as a linker of different co-expression modules. Based on the co-expression of TFs, these 9 novel miRNA-TF pairs potentially involved in plant defense response and plant hormone signaling including auxin, BR, Eth and SA signaling.

Overall, we concluded that the genetic regulatory mechanism underlying the resistance to *C. fulvum* of *Cf-10* carrying line is involved in the widespread upregulation of key genes involved in plant hormone synthesis, signaling pathways and plant-pathogen interaction pathways by downregulating the expression of their regulator miRNAs after invasion. The upregulation of these miRNAs in the susceptible line potentially made it susceptible to *C. fulvum* infection. miRNAs are the key regulators underlying the *Cf-10* gene-mediated defense response to *C. fulvum*.

### 3.7 Quantitative analysis of the major plant hormones verified the genetic regulatory effect of miRNAs on plant hormones

To verify whether the genetic regulation of miRNAs on genes associated with plant hormones caused the hormone level changes at 3 dpi, we quantified the levels of 7 major plant hormones, including auxin, cytokinins (CK), ethylene, ABA, SA, JA, and SL, and 23 of their metabolites in the resistant and susceptible lines for the non-inoculation l and 3 dpi (Supplementary Table S13). For auxin, we quantified the level of indole-3-acetic acid (IAA) and its 10 metabolites. Although we did not observe any changes in IAA, one of the inactive compounds of IAA, 1-O-indol-3-ylacetylglucose, was downregulated by 2.05-fold in the Cf-10-gene-carrying line at 3 dpi (Supplementary Table S13). JA and a precursor of JA, cis(+)-12-oxophytodienoic acid, were downregulated by 3.16-fold in the *Cf-10-gene-carrying line* at 3 dpi, suggesting that the activation of JA synthesis may occur in the period that was earlier than 3 dpi. Jasmonoyl-L-isoleucine (JA-Ile), the metabolite and one of the functionally effective compounds of JA, was upregulated by 2.33-fold in the *Cf-10*-gene-carrying line at 3 dpi (Supplementary Table S13). For SA, both SA and salicylate 2-O-β-D-glucoside (SAG, a storage compound of SA) were increased by 2.56- and 2.64-fold, respectively, in the *Cf-10*-gene-carrying line at 3 dpi, which possibly resulted from the downregulation of miR9472 which had increased the expression level of *SARD1* and therefore enhance the SA synthesis. For CK, both cis-zeatin and the precursor of cis-zeatin, 2-methylthio-cis-zeatin riboside, were upregulated in the *Cf-10*-gene-carrying line at 3 dpi (Supplementary Table S13). Furthermore, we did not observe significant changes in other hormones and their metabolite-*Cf-10*-gene-carrying lines at 3 dpi, including ethylene, ABA and SL, which might be because our investigation did not include enough metabolites for these hormones. Overall, the levels of auxin, JA and CK were changed in the *Cf-10*-gene-carrying line at 3 dpi, where the change in JA levels may be due to the regulation of ILL6 by miR9472.

By comparing the plant hormone level between resistant and susceptible lines at 3 dpi, we observed that an inactive type of IAA, methyl indole-3-acetate, was downregulated in the *Cf-10*-gene-carrying line (Supplementary Table S14). The conjugated product of CK, N6-isopentenyladenine, was downregulated in the *Cf-10*-gene-carrying line. For SA, SA and SAG were upregulated in the *Cf-10*-gene-carrying line. JA, the effective compound of JA, jasmonoyl-L-isoleucine, and an intermediate product of JA biosynthesis, cis(+)-12-oxophytodienoic acid, were upregulated in the *Cf-10*-gene-carrying line at 3 dpi. For other hormones, we did not observe differences between the two lines. In summary, after the invasion of the fungus, the *Cf-10-*gene-carrying line had higher levels of SA and JA but lower levels of CK and IAA, which provided a better resistance ability.

### 3.8 Expressional validation of the miR9472-ILL6-JA signaling pathway

**FIGURE 5:**
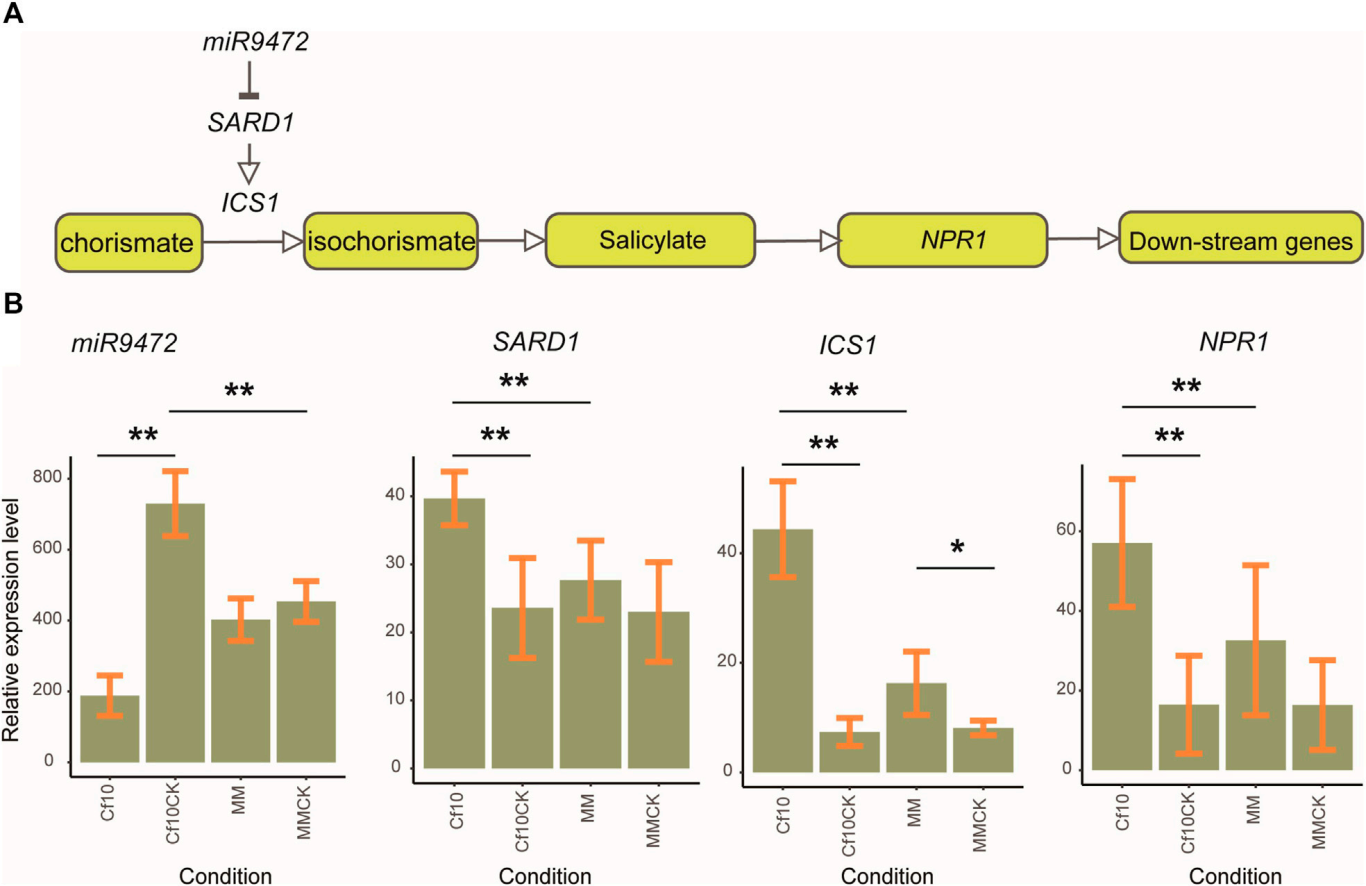
Validating the expression pattern of the miR9472*-SARD1-*SA pathway in resistant and susceptible lines for the non-inoculation and 3 dpi. **(A)**. The relationship between miR9472-*SARD1* and the JA synthesis and signaling pathway. **(B)**. Expression pattern of miR9472, *SARD1*, ICS1 and *NPR1* in resistant and susceptible lines for the non-inoculation and 3 dpi, as revealed by qPCR experiments. **: representing for significance under *p*-value ≤0.01; *: representing for significance under *p*-value ≤0.05.

We further validated the gene expression pattern of the miR9472-SARD1-SA signaling pathway (Figure 5) by real-time quantitative PCR (RT–qPCR). We selected 3 plants of both lines for the non-inoculation and 3 dpi to validate the expression patterns of miR9472, SARD1 and key genes of the SA synthesis and signaling pathway, which showed high expression in our samples, including ICS1 (*Solyc06g071030*) and NPR1 (*Solyc04g040220*). miR9472 showed the highest expression in the non-inoculation of the *Cf-10*-gene-carrying line, followed by the non-inoculation of the susceptible line and susceptible line at 3 dpi, with the lowest level in the *Cf-10*-gene-carrying line at 3 dpi (Figure 5). *SARD1* had the highest expression in the *Cf-10*-gene-carrying line at 3 dpi, followed by the susceptible line at 3 dpi and with the lowest expression in the *Cf-10*-gene-carrying line for the non-inoculation and in the susceptible line for the non-inoculation (Figure 5). ICS1 showed a consistent expression pattern with *SARD1*, which had the highest expression in the *Cf-10*-gene-carrying line at 3 dpi, followed by the susceptible line at 3 dpi and with the lowest expression in the *Cf-10*-gene-carrying line for the non-inoculation and in the susceptible line for the non-inoculation (Figure 5). For *NPR1*, compared to the non-inoculation, we observed that it was upregulated in both the resistant and susceptible lines at 3 dpi, with the former showing much higher expression than the latter (Figure 5). In summary, our expression analysis further supproted that the miR9472-*SARD1*-SA pathway was upregulated in the *Cf-10*-gene-carrying line at 3 dpi compared to its non-inoculation, suggesting that the pathway plays a regulatory role in the defense response of the *Cf-10*-gene-carrying line.

## 4 Discussion

Leaf mold is the most serious disease affecting the reproduction of tomatoes and is caused by infection with the fungus *Cladosporium fulvum.* The *Cf-10* gene has been proven to confer resistance to *C. fulvum*. By performing a multiple omics study of the *Cf-10* gene-carrying line and a non-inoculation line that did not carry any resistance gene for the non-inoculation and 3 days post-inoculation (dpi) with the fungus, our results indicated that miRNAs potentially play regulatory roles in critical genes of defense response pathways that have been reported to respond to the invasion of *C. fulvum*, including plant-pathogen interaction pathways, plant hormone signaling pathways and detoxification processes (Figures 3 and 4). We concluded that miRNAs should play crucial roles in *Cf-10*-mediated disease resistance to *C. fulvum*.

For the plant-pathogen interaction pathway, we observed that miR6026 regulated three important avirulence gene recognition genes, R1B-23, R1A-3 and RPM1 (Grant et al., 1995), with miR6026 downregulated and the three genes upregulated at 3 dpi in the *Cf-10*-gene-carrying line (Figure 4A). R1B-23 and R1A-3 are homologs of the late blight resistance protein Prf, which contains the NB-LRR protein and interacts with the protein kinase Pto to confer recognition of Avr proteins, including AvrPto and AvrPtoB (Salmeron et al., 1996). RPM1 is also an Avr-recognizing gene that interacts with RPM1 interacting protein 4 (RIN4) to recognize the Avr proteins AvrB and AvrRpm (Leister et al., 1996). These results suggested that NB-LRRs were potentially regulated by *Cf-10* gene for triggering downstream immune response. Meanwhile, miR482 regulated the avirulence gene recognition gene Pto (Martin et al., 1993), where Pto interacted with Prf to confer recognition of AvrPto and AvrPtoB as described above. As with Pto, RPM1 and Prf trigger host hypersensitive cell death to prevent the spread of pathogens (Salmeron et al., 1996; Leister et al., 1996), and these results suggested that miR6026 and miR482 work together to regulate the hypersensitive response by targeting RPM1 and Pto/Prf. Furthermore, miR156 regulates the avirulence gene master regulator Upa20, which recognizes AvrBs3 to cause cellular hypertrophy (Kay et al., 2007). miR396 regulates the cyclic nucleotide gated channel gene CNGC, which transduces calcium signaling activated by the invasion of pathogens and then triggers the host hypersensitive response (Moeder et al., 2011). Therefore, we deduced that the *Cf-10*-mediated defense response may occur primarily by activating a host hypersensitive response.

For plant hormone signaling pathways, we observed a decreased level of auxin and increased level of JA, SA and CK (Supplementary Table S13), as well as miRNAs targeting auxin, JA, ethylene, BR, GA and ABA but no SA related genes in this study (Figures 4A,C–G). For auxin, the novel miRNA novel_miR_216 regulates the auxin receptor AUX1 (Bennett et al., 1996). In our analysis of metabolites of auxin, we observed that the inactive type of IAA was downregulated, possibly suggesting that the level of auxin was improved in the *Cf-10*-gene-carrying line at 3 dpi. For the ethylene signaling pathway, downregulation of miR168 and novel_miR_52 upregulated the ethylene receptor ETR2 (Sakai et al., 1998) and response transcription factors EIN4 (Hua et al., 1998) and ERN1 (Trentmann, 2000) (Figure 4D). Although the regulation of ethylene signaling genes by miRNAs has been reported in previous studies, the regulation of EIN4 and ETR2 by miR168 has not yet been identified, suggesting that miR168 may play a role in the ethylene pathway during the defense response. For BR and GA, miR156, miR482 and the novel miRNA novel_miR_250 regulated BR receptor BRI1 (Nam and Li, 2002) and BR signaling transducer BSK (Sreeramulu et al., 2013), and novel_miR_272 and novel_miR_64 regulated the key GA response transcription factor GAI (Silverstone et al., 1997). Consistent with a previous study that reported that miR156 was induced by BR treatment, miR156 may play a regulatory role in BR signaling during the defense response (Xie K et al., 2012). However, except for auxin, we did not observe a difference in these hormones between the non-inoculation and 3 dpi in our plant hormone and hormone metabolite analysis. This result may be because our metabolism analysis did not include enough metabolites for these hormones, or the difference in these hormones between the non-inoculation and 3 dpi *Cf-10*-gene-carrying line could only be detected at other time points, not at 3 dpi. In summary, we concluded that miRNAs regulated receptors or key signaling genes of auxin, ethylene, BR and GA in the *Cf-10*-mediated defense response.

Studies have reported that miR9472 responds to various abiotic and biotic stresses (Candar-Cakir et al., 2016; Sharma et al., 2021), but the underlying regulatory mechanism is not yet understood. In our study, we showed that miR9472 negatively regulated *SARD1* (Figure 4H; Figure 5), where miR9472 was downregulated and *SARD1* was upregulated at 3 dpi. Expression validation of the miR9472-SARD1-SA pathway further supproted that their expression was improved in the *Cf-10-*gene-carrying line at 3 dpi compared to the corresponding non-inoculation (Figure 5). *SARD1* has been reported to be a a key regulator for ICS1 (Isochorismate Synthase 1) induction and salicylic acid (SA) synthesis which improved the SA level after responding to the invasion of bacterium. Our results indicated that miR9472 could potentially regulated SA synthesis and signaling by targeting to *SARD1*, where the downregulation of miR9472 improved the expression of its target *SARD1* to improve the level of active SA compounds, therefore enhancing the JA signaling pathway to improve the host defense response.

In addition, miR9472 also negatively regulated ILL6 (Figure 4G and Figure 5) (Widemann et al., 2013), where miR9472 was downregulated and ILL6 was upregulated at 3 dpi. ILL6 has been reported to mediate the turnover of major active component (+)-7-iso-jasmonoyl-l-isoleucine (JA-Ile) of JA to JA and Ile to reduce effective JA level (Widemann et al., 2013). But in our quantitative analysis of major plant hormones and their metabolites (Supplementary Table S13), we observed that the level of JA-Ile was improved in the *Cf-10*-gene-carrying line at 3 dpi compared to its non-inoculation.This unusual phenomena has been reported by previous study. However, it is still unknown whether this effect could enhance the JA signaling or improve JA level by feedback.

We observed that a large proportion of pathogen-responsive miRNAs played a negative role in the defense response of the *Cf-10*-gene-carrying line: without pathogen infection, the *Cf-10*-gene-carrying line expressed abundant miRNAs, such as miR156, miR396 and miR6026, possibly triggering miRNA-induced gene silencing to reduce the abundance or suppress defense response genes, but after *C. fulvum* infection, these miRNAs were downregulated to simultaneously activate defense response genes. One possible explanation for this phenomenon is that the fine-scale regulation of defense response genes by miRNAs would alleviate the host energy waste to maintain the potential of resistance to pathogens under normal conditions but could quickly activate defense response pathways after the invasion of pathogens by suppressing the expression of miRNAs. A similar strategy has been frequently reported in plant responses to abiotic stress (Huot et al., 2014; Karasov et al., 2017), which are generally also involved in antagonistic crosstalk among hormones and the conditional specific regulation of R genes. However, only a few of them have reported the negative role of miRNAs in coordinating plant immunity and normal growth (Salmeron et al., 1996). Further studies that clarify the explicit role of miRNAs in coordinating plant immunity and growth will improve our understanding of how to modulate them to enhance the defense response and maintain high-level reproduction.

## 5 Conclusion

In this study, by multiple omics profiling of tomato lines with/without *Cf-10* genes before inoculation and 3 dpi and 16 dpi of *C. fulvum*, we demonstrated 54 miRNAs that were differentially expressed in the non-inoculation and 3 dpi *Cf-10*-gene-carrying line, as well as 68 differentially expressed miRNAs between the resistant and susceptible lines at 3 dpi. In silico prediction yielded 894 target genes for the 54 miRNAs differentially expressed in the non-inoculation and 3 dpi *Cf-10*-gene-carrying line and 792 target genes for 68 differentially expressed miRNAs between the resistant and susceptible lines at 3 dpi. Combining differentially expressed miRNAs and DEGs indicated that crucial avirulence gene recognition genes and signal transduction genes in the plant-pathogen interaction pathway and the receptors/key response transcription factors (TFs) of plant hormone signaling pathways were negatively regulated by miRNAs, where these genes were generally suppressed by miRNAs under normal conditions but were activated by downregulating the expression of miRNAs. Therefore, the resistant line employ a “trade-off” strategy to coordinate growth and defense responses (Supplementary Figure S6): without pathogen infection, they express abundant miRNAs to suppress defense response genes, but after *C. fulvum* infection, they downregulate these miRNAs to simultaneously activate many defense response pathways to enhance resistance, therefore coordinating the expression cost of defense response genes and plant growth.

## Data Availability

The datasets presented in this study can be found in online repositories. The names of the repository/repositories and accession number(s) can be found in the article/Supplementary Material.
