# Supplementary figures and images for: Integrating omics reveals that miRNA-guided genetic regulation on plant hormone level and defense response pathways shape resistance to Cladosporium fulvum in the tomato Cf-10-gene-carrying line
Source: Front Genet. 2023 May 25;14:1158631. doi: 10.3389/fgene.2023.1158631 (PMC10248068; doi:10.3389/fgene.2023.1158631)

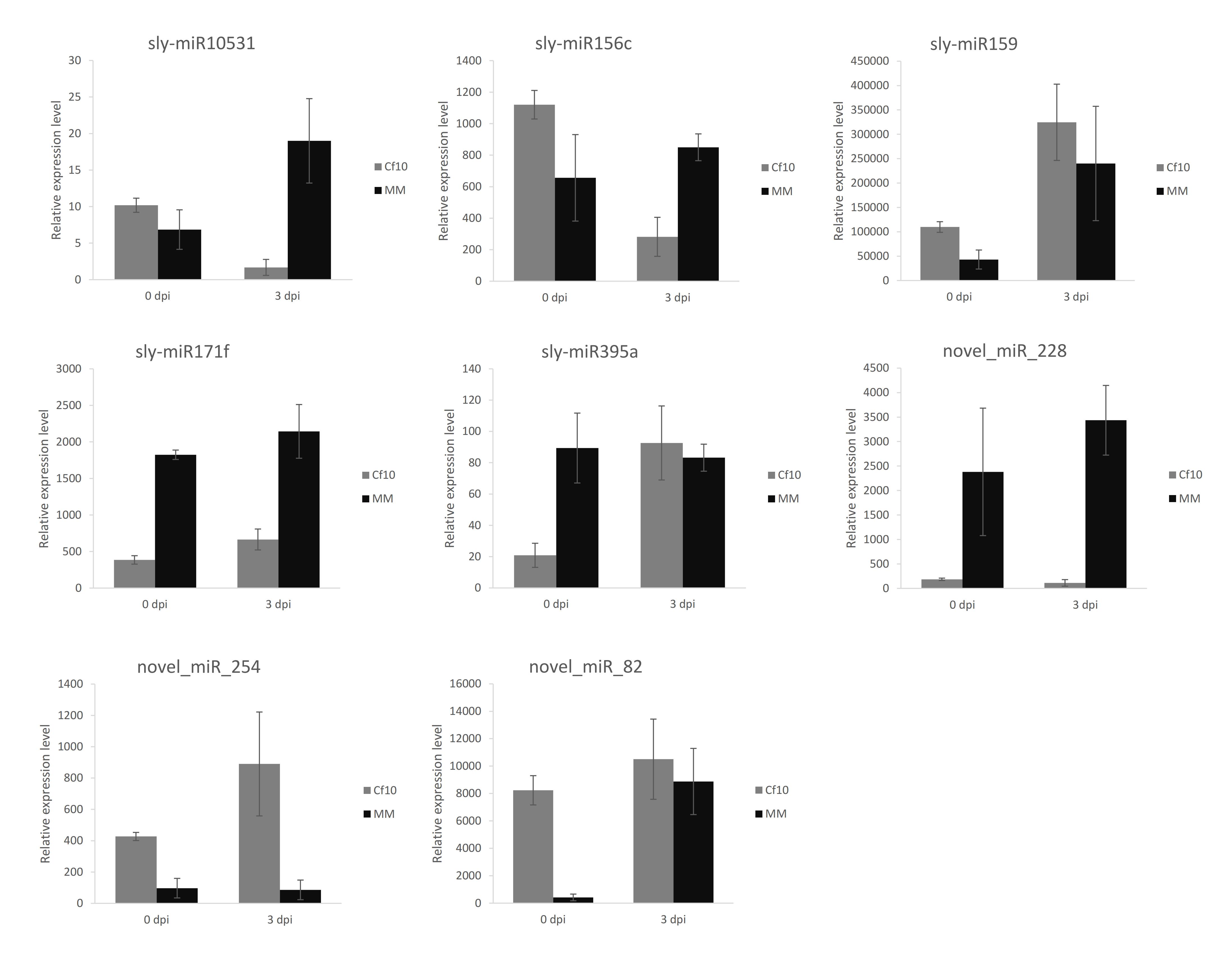

Supplement: Supplementary file 3 [file Image3.JPEG]

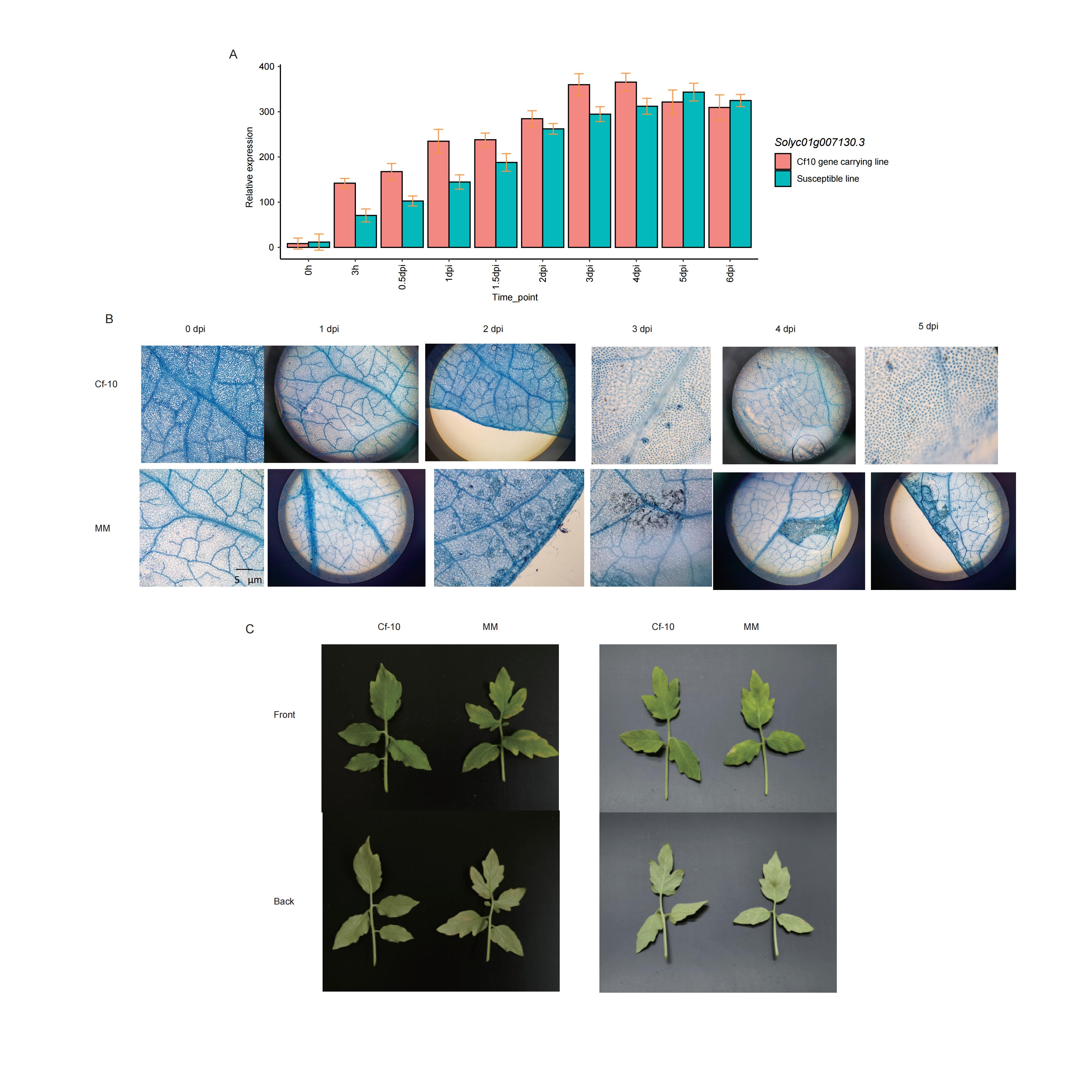

Supplement: Supplementary file 6 [file Image1.JPEG]

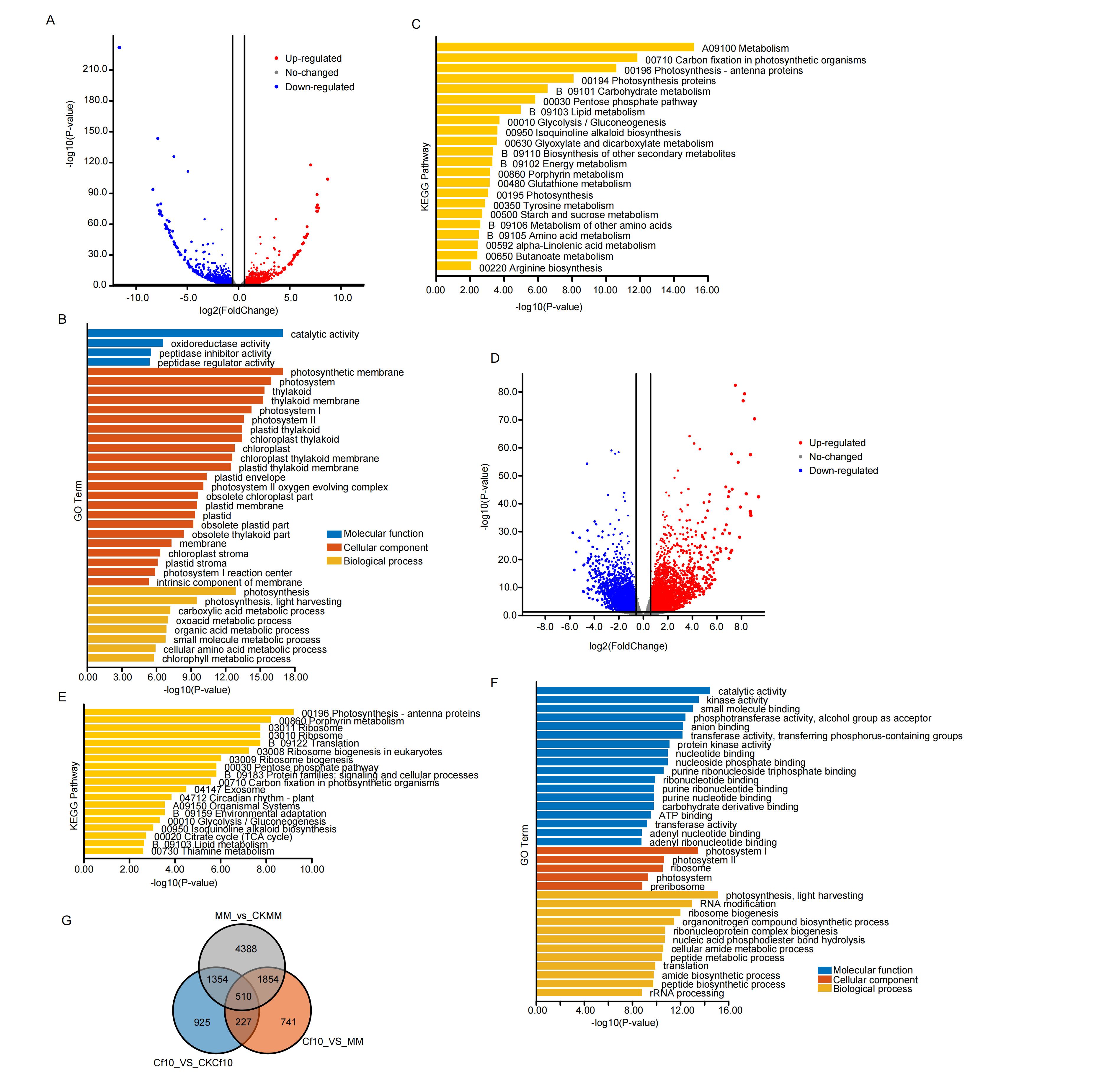

Supplement: Supplementary file 7 [file Image4.JPEG]

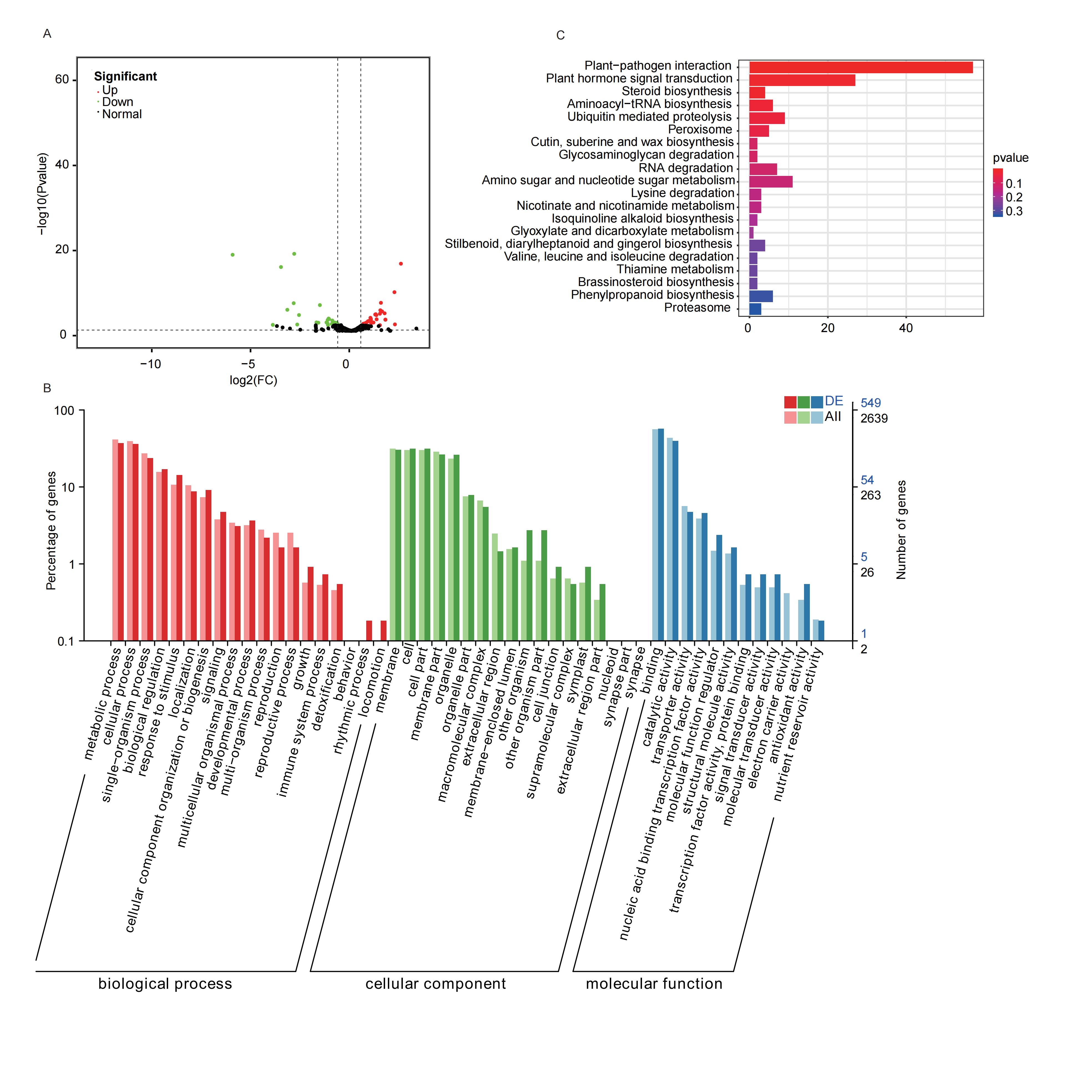

Supplement: Supplementary file 8 [file Image2.JPEG]

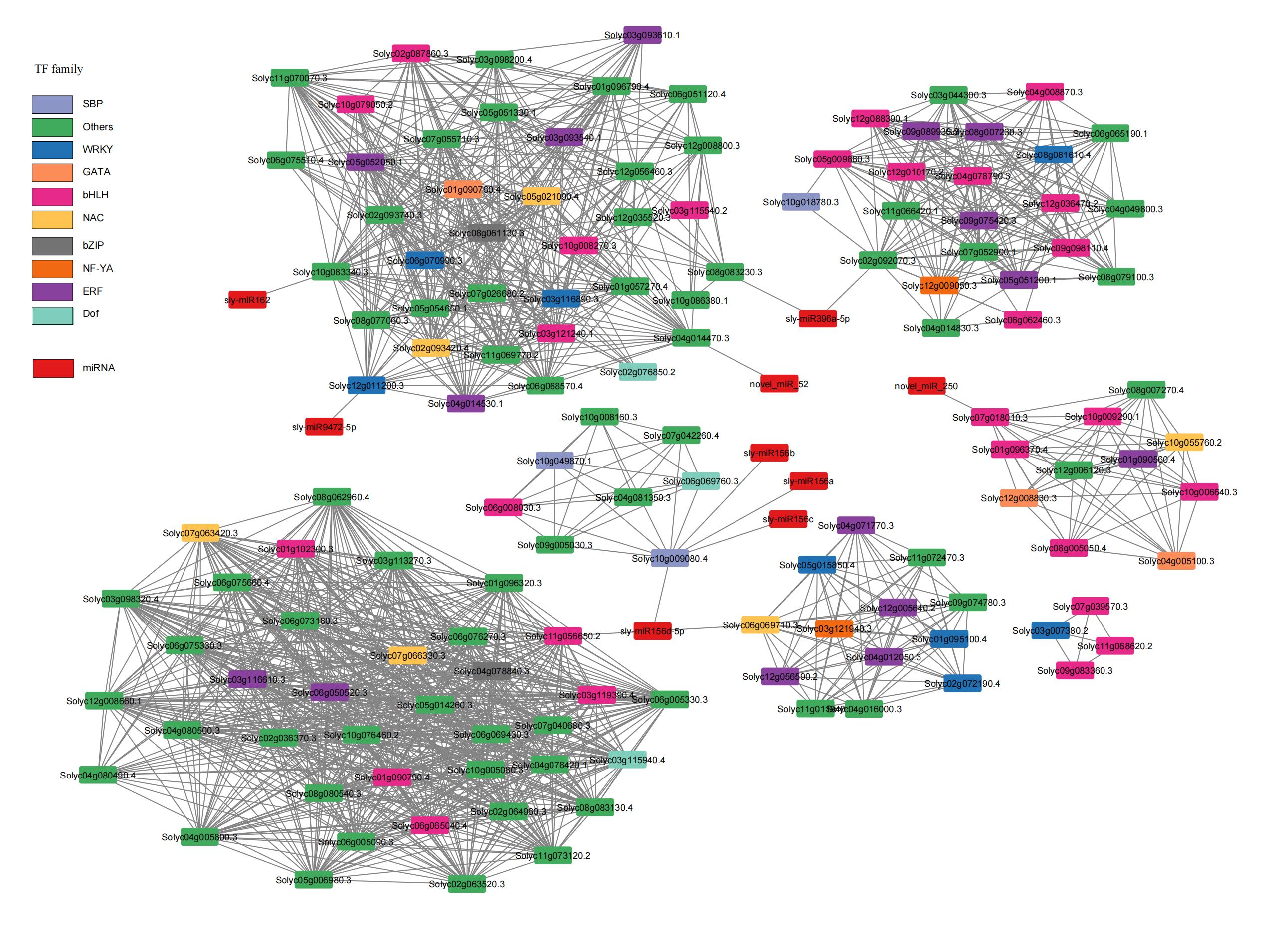

Supplement: Supplementary file 9 [file Image5.JPEG]

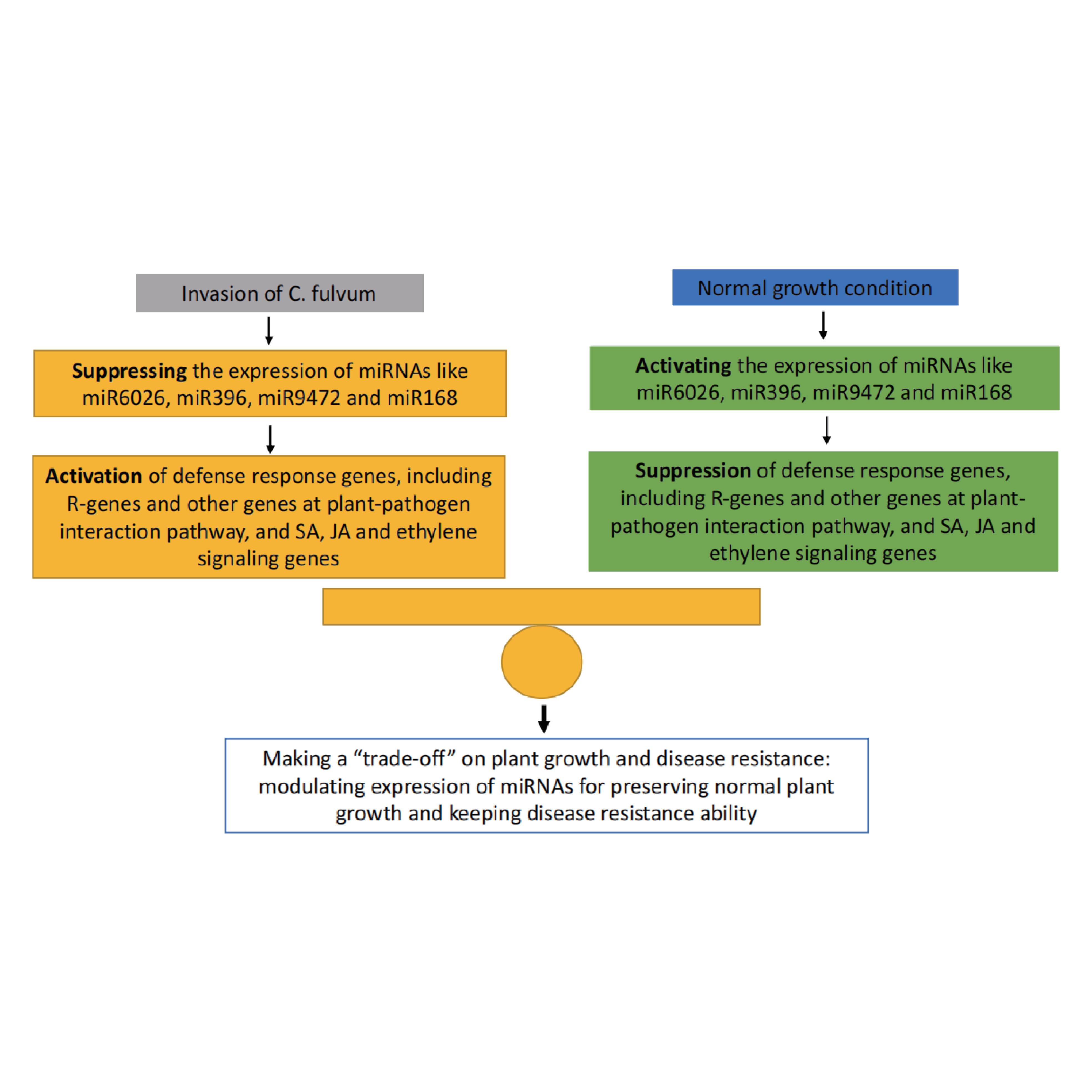

Supplement: Supplementary file 21 [file Image6.JPEG]
